# New visual culture of science: analysis of graphical abstracts in the life science journal “*Cell*”

**DOI:** 10.1007/s40656-026-00763-0

**Published:** 2026-09-16

**Authors:** Kana Ariga

**Affiliations:** College of Arts and Sciences, J. F. Oberlin University, Tokyo, Japan

**Keywords:** Graphical abstract, Visual culture of science, Scientific images, Epistemic virtue, Life science

## Abstract

Driven by the shift towards online journal publishing, many academic journals have introduced graphical abstracts (GAs) since the 2000s to convey the primary findings of research papers. This study investigated this visual culture in life sciences by analyzing GAs with focus on content, representation, and background. In total, 333 GAs were collected from articles published in the journal *Cell* in 2021 and analyzed. Among these, 295 GAs (88.6%) explained the results and models and succinctly presented the key findings, and 251 GAs (75.4%) used a layout that divided the area into two or more sections. New graphics that were not present in the main text were featured in 222 GAs (66.7%), indicating substantial investment in their creation. The GAs shared commonalities with modern infographics commonly seen on the internet, which emphasize clarity. The increased used of GAs may be attributed to the need for “instant comprehension,” which is an epistemic virtue reinforced by authors, readers, and journals and driven by the scientific self of “surviving researchers and journals” in a competitive scientific environment.

## Introduction

A significant change in recent years in the visual culture of science is the digitization and proliferation of online journals. Historically, changes in media have brought new visual culture to science. For example, the printing technology of the 16th century notably changed the manner of dissemination of scientific knowledge by delivering illustrations and figures to many readers without concerns of errors in reproduction (Ivins, 1953). However, the impact of digitization extends beyond making articles available for viewing on websites when previously they were only accessible in print. Digitization has increased accessibility, number of citations, and searchability through search engines; additionally, online articles are equipped with functions such as animation, audio, and hyperlinks that cannot be found in print media. Furthermore, the elimination of print media has reduced costs and increased the number of journals published (International Association of Scientific, Technical & Medical Publishers, 2021).

This study focuses on the graphical abstract (GA), which is a new type of image that has become prominent in the era of these media changes. GAs are single concise visual summaries of research articles. These are alternatively known as tables of content (TOCs), visual abstracts, or infographics. They are featured on article list pages and/or on article pages of online journals and are often used for dissemination via social media. By conveying a summary of the article in the GA, the journal or authors aim to enhance interest in the article, assist readers in readily identifying articles that are worth reading, and ultimately contribute to citation and funding acquisition (Cox, 2015; Ramos & Concepcion, 2020).

According to Lane et al., (2015), GAs originated in 1976 and first appeared in the German chemical journal *Angewandte Chemie* during the era of print media. However, many academic journals have adopted GAs with the progress of transition to online journals in the 2000s. For example, GAs appeared in social sciences in 2010 and their usage increased by 350% between 2011 and 2015 (Yoon & Chung, 2017). Currently, GAs are widespread alongside digital publishing technologies.

Thus, GAs may constitute an emerging visual culture of science. Hentschel, (2014) has noted that visual culture emanates from small communities in science shaped by devices, practices, and technologies, and its existence is contingent on the background of the era and other context. The study of visual culture in science is not confined to image analysis but rather identifies characteristics in a series of visual practices such as recognition, thinking, training, acceptance, and image utilization. Therefore, even a single type of image such as GAs may create a new visual culture that reflects the changes in the perceptions and practices of scientists.

This study investigates the characteristics of GAs in life sciences with specific focus on the type of information represented and manner in which it is conveyed. Furthermore, the intersection of social and scientific contexts have been analyzed to understand the underlying reasons for the widespread adoption of GAs. Finally, this study aims to elucidate the distinctive features of the visual culture emerging from this medium.

## Literature review

Previous studies on GAs have garnered significant attention regarding the influence of GAs on papers in terms of the number of citations, visibility, and number of downloads. However, some studies have indicated a lack of correlation between GAs and number of citations or article usage; instead, they suggest that GAs only enable first attention (Pferschy-Wenzig et al., 2016; Zong et al., 2023). In contrast, study on the impact of GAs on social media has shown largely positive outcomes. Several reports indicate that GAs have enhanced the impact of articles on social media and increased engagement metrics such as impressions and retweets (e.g., Lindquist & Ramirez-Zohfeld, 2019; Oska et al., 2020; Chisari et al., 2021). Additionally, journals that adopted GAs between 2019 and 2021 have reported an increase in their impact factors (Kim et al., 2022). These studies reflect the expectations of scientists that GAs would enhance the social impact of their work, which includes increased citation and page views.

Hendges and Florek (2019) have stated that GAs use the visual semiotic mode to convey meaning and package information in a manner that enables the entire picture to be captured in an instant. Other studies and articles that summarize the key factors in creating GAs have provided design tips such as “having a clear take-home message” and “avoiding using too much text” with the aim of guiding scientists in creating GAs (e.g. Ibrahim, 2018; Ramos & Concepcion, 2020; Kitterman, 2021; Spicer & Coleman, 2022). Hullman & Bach (2018) studied GAs on aspects of design and representation and classified them into eight types of layouts, four types of time expression, five types of text use, and six types of expression genres. Sancho-Guinda, (2022) classified GAs according to meaning and expression into narrative, classificatory, zoom-in, and data display categories, while positing that GAs function to increase research impact through comprehension-assisting designs. These studies further suggest that GAs possess unique representational and stylistic conventions. Consequently, this study seeks to define the essential features of GAs by elucidating the prevailing trends in their design and content.

Nevertheless, GAs may be related to images traditionally used in research papers and textbooks. Maienschein (1991) noted that in the E. B. Wilson textbook *The Cell*, the first edition presented empirical images that represented “facts,” whereas the third edition had shifted toward diagrammatic illustrations that represented theoretically driven interpretations. Maienschein attributed this transition to factors such as the author’s (Wilson) increasing certainty about his observations, increasing volume of knowledge, and greater sophistication on the author’s part in recognizing the pedagogical and heuristic value of diagrams. Consequently, both textbooks and the latter parts of research papers tend to prioritize interpreted schematics over objective photographs. Regarding these diagrammatic illustrations in life sciences, Abrahamsen et al., (2018) have noted that most mechanism diagrams comprised different kinds of elements called glyphs. This raises the question of whether the representational characteristics of GAs in life sciences align with those diagrams and their usage trends. In fact, Hendges and Florek (2019) have noted that some GAs were reproduced from figures in the papers. Moreover, scientific visual images may be influenced by artistic conventions of the time (Rudwick, 1992). Kemp (2000) argues that art and science do not merely interact but share a common structure based on the human quest for visual understanding. Thus, to gain a deeper understanding of GAs, their similarities and differences must be considered not just relative to images in scientific papers and textbooks but also relative to their relationship to visual representations in the field of art.

Daston and Galison (2007) have identified three epistemic virtues in modern science by analyzing atlas images and examining their connections to the scientific self. They argue that the epistemic virtue in the 18th century was “truth-to-nature,” and the scientific self in that virtue was a sage who succeeded in finding the essential and ideological forms of specimens. However, this approach was criticized as subjective in the mid-19th century and was replaced by the virtue of “mechanical objectivity,” which avoided intervening in the atlas as much as possible and sought to depict the images as they were. This view calls for the scientific self to be a worker who has renounced will. In the 20th century, the epistemic virtue of “trained judgment” emerged, and it sought to train the viewer’s skills to interpret images. In this scenario, the scientists establish their scientific selves as experts. However, the division of history into these three distinct periods has been subjected to criticism with the argument that the first and third of these three periods are not clearly distinguishable and that the third stage was as much in effect during the foregoing two periods (Hentschel, 2008). Moreover, questions have been raised as to whether scientists in the era of mechanical objectivity were in fact fervently acting to negate his or her own presence (Anderson, 2009). Nevertheless, the perspective that the scientific self and epistemic virtues within specific historical contexts shape the practices of scientists is considered a useful framework for understanding science. Hence, GAs may be considered a new type of atlas to help identify the kind of epistemic virtue or scientific self that they represent.

This study investigates the characteristics and underlying factors of the new visual culture in science that has emerged in the 21st century. To this end, this study examined these characteristics by analyzing representation and content in GAs.

## Analysis

### Analysis subjects

GAs are used in various fields including chemistry, life sciences, medicine, and environmental sciences. Their representative styles vary depending on the field. This study focused on the GAs published in *Cell*, which is a leading journal in the life sciences field. *Cell* is an internationally peer-reviewed journal published by Cell Press (Elsevier). It was first published in 1974 and is recognized as a top global scientific journal. Scientists in the life sciences fields traditionally use diagrams and illustrations in academic discussions. As images used in life science and those published in *Cell* have been analyzed in previous studies (Ariga & Tashiro, 2022), a comparison could be made between the characteristics of GAs and life science images not appearing in GAs.

### Analytical method

This study analyzed the guidelines for GAs and their placement in addition to performing an empirical analysis of the GAs themselves. This study investigated the expectations imposed by the journal by examining the guidelines of *Cell* and the positioning of GAs in the website and PDF versions of articles. Additionally, all 333 GAs were selected from articles labeled “Research article” on the ScienceDirect *Cell* journal, Volume 184, Issues 1–26, published in 2021. The selected GAs were classified based on their characteristics such as the type of content, layout, representation, and correspondence with the figures in the articles. The classification categories (described later) were formulated with reference to previous studies and after observing the sample GAs. These results and those from previous studies were combined to infer the visual culture of GAs.

## Results

### GAs in *Cell*

What specific roles do the journals expect GAs to fulfill? A crucial factor to consider regarding the role of GAs is the readers’ perception of the GA. Cell Press displays GAs in *Cell* as small thumbnails in the list of articles for each issue; however, the contents of the images are difficult to discern in this form. In contrast, the *Cell* issue page on ScienceDirect lists the article titles, authors, and PDFs without displaying the GAs. Furthermore, GAs are not included in PubMed pages, although it is a database that is frequently used by life science scientists for literature searches. This suggests that GAs may not play a significant role in identifying the papers to read.

In terms of positioning of the GAs on the article page, the *Cell* article pages on both Cell Press^1^ and ScienceDirect^2^ follow a similar structural flow from the top with bibliographic information such as page number, title, author names, highlights, summary, GA, keywords, and main text. Consequently, while scrolling down, the readers first see the title, author names, highlights, and summary, followed by the GA before reaching the main text.

This layout suggests that readers who follow the order of the page first understand the theme and overview of the paper through the text; then, they encounter the GA immediately before delving into the main text. The expected role of the GA in this context is to provide a visual summary of the major study points after the reader has grasped the textual overview. Thus, the GA acts as an intermediary step before the readers read the detailed content. Although some readers may skip or read out of order, the positioning implies that the GA helps readers to quickly understand the gist of the article and decide whether or not to read the full text.

Next, the instructions provided by the journals to authors regarding GA creation were analyzed. According to the Cell Press GA guidelines^3^, a GA is defined as a single-panel image designed to provide readers with an immediate understanding of the take-home message of the article. The recommended size is 1200 × 1200 pixels at 300 dpi with Arial font at sizes ranging from 12 to 16 pt. Additionally, the colors used need to match or complement those on the Cell Press website. For clarity, the guidelines suggest that the start and end points of the image be indicated to make it readable from top to bottom and left to right. The GA is expected to visually represent the biological context of the results, distinguish itself from model diagrams or figures in the article, emphasize new findings without delving excessively into previous literature, avoid speculative elements, exclude data items of any type, and be in a graphical form. Additionally, the authors were advised to keep the design simple, clearly highlight one process or point, and eliminate distracting elements.

These guidelines are intended to enhance the visual clarity and accessibility of GAs and provide the necessary information with focus on key study points to facilitate understanding. These efforts align with the overarching objective of enabling readers to grasp the core message of a paper instantaneously.

Among the GAs included in this study, some authors strictly adhered to these guidelines, whereas others did not. In fact, non-compliance with the guidelines was observed in several instances such as the use of small font sizes (e.g., Karki et al., 2021; Obradovic et al., 2021) and color schemes that neither matched nor complemented the signature blue of the Cell Press website (e.g., Wang et al., 2021a, b; Sposito et al., 2021, which lacked the characteristic light blue or analogous tones). Furthermore, some GAs used non-linear layouts such as the circular design (e.g., Doron et al., 2021) instead of the standard top-to-bottom orientation. Additionally, in some cases, the figures from the main text were reused (discusses later).

Thus, adherence to these guidelines is not strictly enforced, and deviation does not necessarily prevent publication. These deviations may be attributed to either the insufficient understanding of the guidelines by the author or deliberate decision to prioritize their own preferred modes of expression at the expense of compliance (for example, using smaller text to include more information). As noted by Hullman & Bach (2018), effectively implement such guidelines may be challenging for scientists because they typically have little formal training in graphic design. In this scenario, deviating from the guidelines may reduce the authors’ workload but is unlikely to provide substantial benefits for readers or journals.

### Content included in GAs

**Fig. 1:**
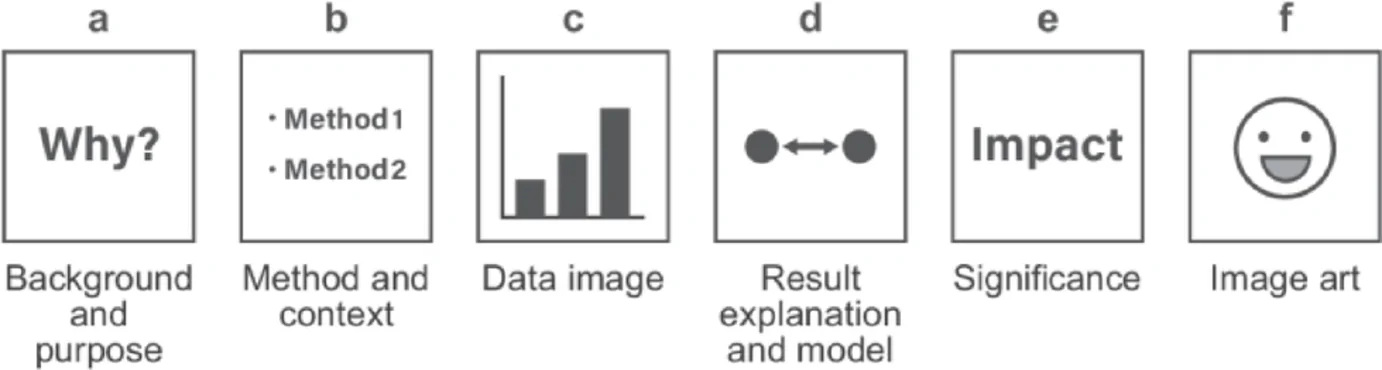
Six categories of content in GAs

Generally, academic papers in life sciences contain information such as background, purpose, methodology, results, and discussion. Based on these general trends, the guidelines for GAs in *Cell*, and analysis of the GAs, the information in the GAs are classified into the following categories (Fig. 1):

1. * Background and purpose*: Context and status of relevant studies, problem statement, and research objectives
2. *Methods and context*: Research methodology and biological context related to the research subject (e.g., species and cell types).
3. * Data image*: Images showing results such as data visualization and microscopy images.
4. * Result explanation and model*: Explanation of result interpretation, new findings, theories, and models.
5. * Significance*: Impact and effect on academia and society.
6. * Image art*: Eye-catching pictures that do not express scientific information directly or accurately.

The GA contents were counted based on this classification. For example, a GA containing multiple graphs was counted as containing one “Data image” content. GAs that included images related to both “Methods and context” and “Data image” were counted as containing one “Methods and context” and one “Data image” content.

**Fig. 2:**
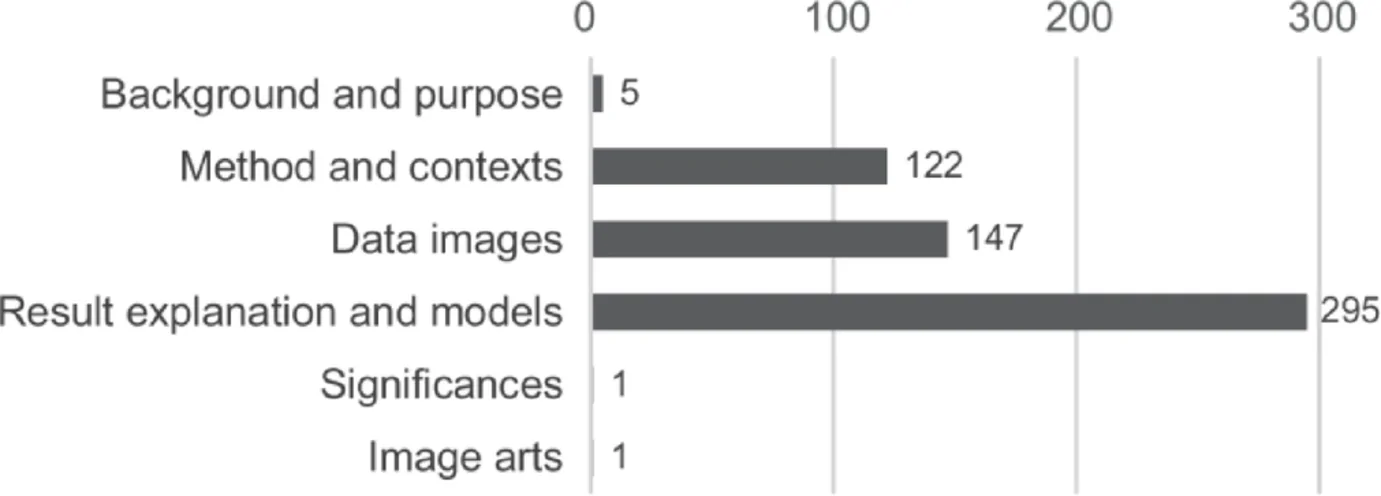
Number of the contents in GAs

**Table 1:** Combination of contents in GAs

Contents	Number	Percentage
Result explanation and model (only)	132	39.6%
Data image/Result explanation and model	60	18.0%
Method and context/Data image/ Result explanation and model	49	14.7%
Method and context/Result explanation and model	47	14.1%
Method and context/Data image	21	6.3%
Data image (only)	16	4.8%
Others	8	2.4%

**Fig. 3:**
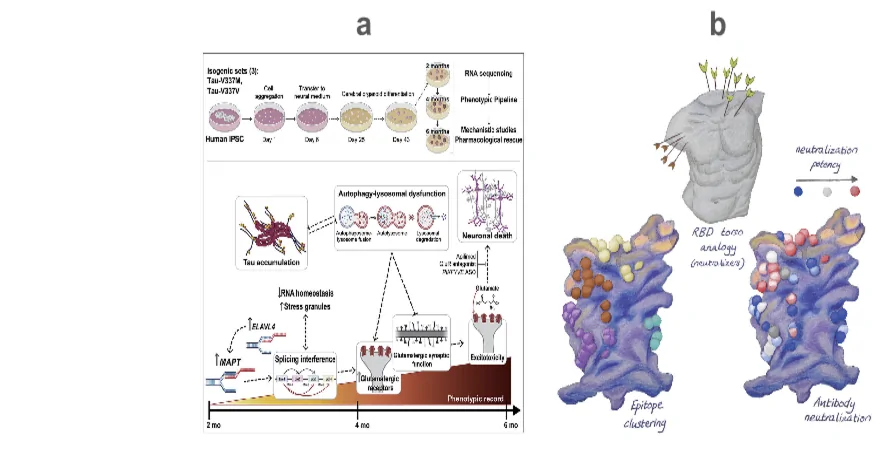
Examples of the contents in GAs.** a** This GA summarizes the early alterations because of the mutation that preceded neurodegeneration and identified through experiments using MAPT-mutant cerebral organoids (Bowles et al., 2021). The upper panel of this GA details the “Method and context,” whereas the lower panel illustrates the “Result explanation and model.”** b** This GA represents the antigenic structure of the SARS-CoV-2 receptor binding domain (Dejnirattisai et al., 2021). The illustration at the top is an “image art” using analogy and is not intended to be a precise representation of scientific structural data

The results after classification are presented in Fig. 2; Table 1. As mentioned earlier, Sancho-Guinda (2022) classified GAs according to meaning and expression into four categories; however, the results suggest that the GA content appears to be combined in more complex ways than that defined by this typology. Most GAs include the “Result explanation and model,” which aligns with the journal’s directive to facilitate quick comprehension of primary findings. Additionally, several GAs combine “Method and context” and/or “Data image” with the “Result explanation and model” (e.g., Fig. 3a). The high proportion of “Method and context” is likely influenced by the Cell Press GA guidelines, which emphasize that the biological context be provided. Furthermore, the inclusion of supporting information on the primary claims appears to be aimed at helping readers understand the overall logic of the articles.

In contrast, “Background and purpose,” “Significance,” and “Image art” are seldom included in most GAs. Notably, researchers rarely highlight the study’s significance despite it being crucial for funding applications and sponsor reports. This may be because the readers of the journal comprise fellow researchers within the journal community rather than sponsors. This suggests that researchers prioritize connecting through the content of their work rather than its significance. Therefore, the data support the view that GAs are designed primarily to convey the content of the paper rather than elicit empathy within a journal community.

An image art blends artistic elements and emphasizes impact and visual appeal over scientific accuracy, and this is the common approach in general science magazines. Therefore, these promotional or advertisement-like GAs may help attract attention in a manner similar to general-interest publications; however, only one GA in the sample set contained image art. This GA used a metaphorical representation of the Fab-antigen structure, which the study focused on, and conveyed scientific meaning when viewed in conjunction with the other image elements (Fig. 3b). Hence, the role of this GA appears to be partly explanatory rather than merely an eye-catching image. These findings suggest that GAs place greater emphasis on conveying the content of the paper and that the appeal of scientific meaning is more likely to attract the interest of journal readers than solely visual attractiveness.

Graphs were the most common type of “Data image” in GAs. Unlike graphs within articles, the graphs within GAs often omitted axis values and were simplified or abstracted to act as a visual language that concisely conveyed their meaning (e.g., graphs in the GA of Brouwer et al., 2021). This approach may be intended to guide those readers who wish to verify the validity of the data in the main text of the paper.

### Graphics as information panels

The GAs in the sample set often adopted a layout with space divided into panels based on meaning. Information on methods, results, and models was presented within each panel. Additionally, some previous articles explain how to design GAs by dividing the space into sections such as methods and results (Ramos & Concepcion, 2020; Kitterman, 2021; Misiak & Kurpas, 2023). In another study, each panel is referred to as a “graphic,” which indicates a unit of images (Ariga & Tashiro, 2022). Subsequently, the number of graphics included in the GA and the reasons for dividing the space were analyzed.

Researchers have used various visual cues to indicate the boundaries of the graphics. These include background colors, boundary lines, text headings, and numbers or letters that indicate the order and margins between graphics. In the absence of these cues, the GAs were considered “not divided.” In cases where the GAs contained seven or more graphics, the elements were so detailed that they were treated as one graphic with multiple elements. In cases where graphics were nested within other graphics, only the topmost graphics were counted. The results for the number of graphics in GAs are presented in Table 2.

**Table 2:** Number of graphics in GAs

Number of graphics per GA	Number of GAs	Percentage(GAs)
One graphic	82	24.6%
Two graphics	103	30.9%
Three graphics	77	23.1%
Four graphics	48	14.4%
Five graphics	19	5.7%
Six graphics	4	1.2%
Total number of GAs	333	
Total number of graphics	830	

**Fig. 4:**
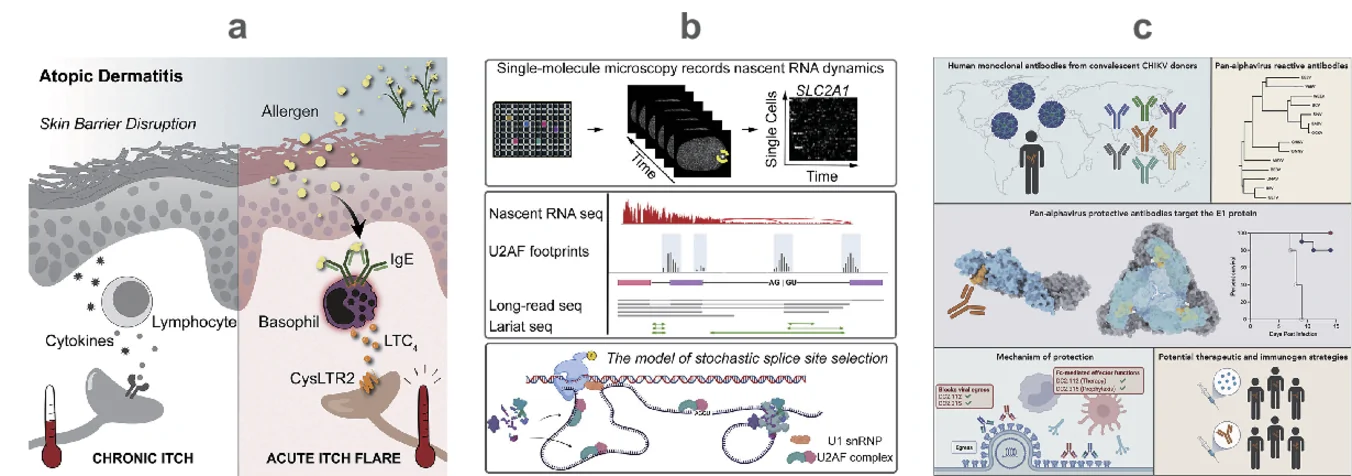
**a** This GA is an example of including two graphics (Wang et al., 2021a, b). The GA illustrates the fundamental itch mechanisms, and a comparison between the left and right graphics shows that the basophil-leukotriene axis plays a critical role in acute itch flares.** b** This GA comprises three graphics. It summarizes how dynamic imaging of nascent RNA has identified the general principles of transcriptional dynamics and stochastic splice-site selection (Wan et al., 2021). The first graphic outlines the experimental methodology, the second presents the results, and the final graphic illustrates the proposed model.** c** This GA comprises five graphics and illustrates how pan-alphavirus protective human antibodies target conserved E1 protein epitopes (Kim et al., 2021). The GA explains that the E1 protein is a target for therapeutics and possible vaccine design by presenting five topics

The results show that 103 (30.9%) GAs contained two graphics. A common approach in this type of layout was the division of the space vertically or horizontally, which enables a comparison between the left and right or upper and lower portions. These contrast-type GAs often present differences in molecular behavior, structural variations, and changes in data under different conditions (Fig. 4a). Furthermore, 77 (23.1%) GAs contained three graphics and often used a story flow pattern. For example, some GAs provided methodological explanations in the first graphic, additional details or data in the middle graphic, and conclusions with models or key findings in the final graphic (Fig. 4b). GAs with four or more graphics were more complicated than those with two or three graphics owing to the inclusion of multiple methods, data images, and/or models (Fig. 4c).

Several researchers used contrast-type GAs possibly because comparative analysis under different conditions (e.g., comparing wild-type and genetically modified types) is a standard research approach in life sciences. Additionally, authors tended to present several data images in one article (Ariga & Tashiro, 2022); hence, multiple datasets may be required to endorse one argument. Moreover, for the readers to understand a claim, it may need to be supported by a logical structure from method to discussion. Thus, a multiple graphics-type GA may have been selected to include multiple types of information into a single GA.

In contrast, 82 (24.6%) GAs contained only one graphic. The type of GA often fell under the “Result explanation and model” category (e.g., Wilde et al., 2021). These images were simple, easy to understand, and impactful because the eyes of the readers were focused on one place. Hendges and Florek (2019) have stated that in a survey of five editors and eleven authors/readers, seven (43.7%) participants indicated that the GA should contain a single image, whereas nine (56.2%) indicated that it should contain a combination of images. Thus, opinions on the most suitable GA type may differ among researchers.

Typically, figures are placed relatively freely within the main text in life science textbooks, and graphics are often placed close to the corresponding text. In such situation, multiple graphics need not be included within a single figure space. In contrast, GAs are presented away from the main text and need to convey the essence of the entire research paper using only a single image regardless of the volume of information. Hence, the GA layout may necessitate multiple divisions to accommodate different graphics to include multiple arguments. This tendency to divide the areas aligns with the conventions observed for presenting figures in the papers. However, the research articles in *Cell* commonly present different graphics within a single figure often by simply juxtaposing the graphics of the divided figure. In contrast, the graphics in the GA are often connected by visual cues. This suggests that the semantic connection of graphics in GAs are stronger than those of the graphics in the article figures.

### GA layouts

**Fig. 5:**
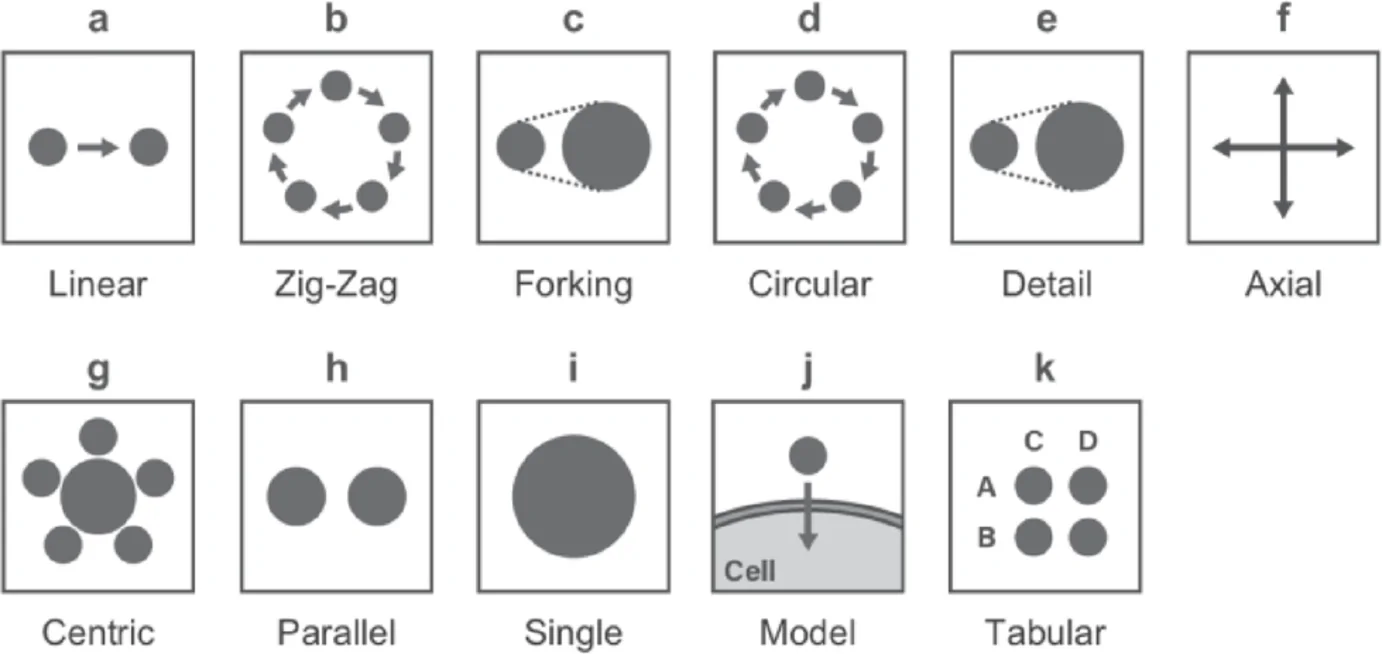
Eleven categories of graphics layouts

**Fig. 6:**
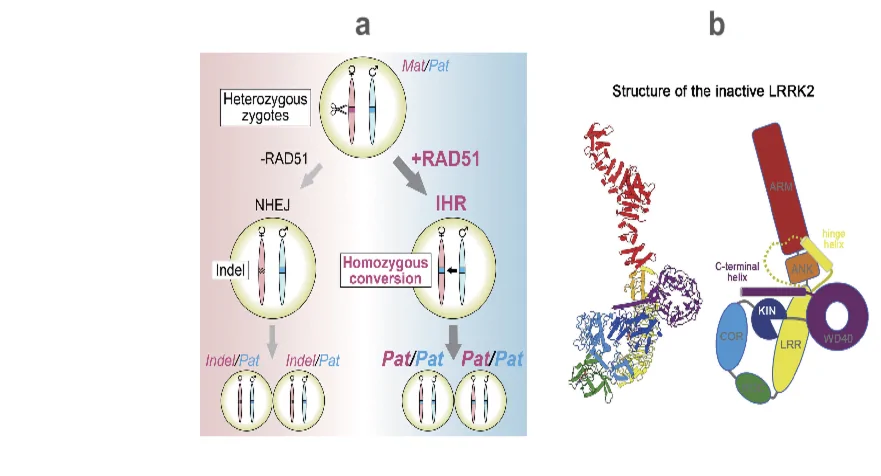
**a** This GA is an example of “forking layout” graphic (Wilde et al., 2021). It presents a comparison between forking lines, which highlights efficient embryonic homozygous gene conversion via RAD51-enhanced interhomolog.** b** This GA uses a parallel layout to present the structural analysis of leucine-rich repeat kinase 2(LRRK2), which is implicated in the pathogenesis of both familial and sporadic Parkinson’s disease (Myasnikov et al., 2021). It features 3D structural models alongside schematic diagrams for direct comparison

Hullman and Bach (2018) collected data on 54 GAs and classified their layouts into eight types: linear, zigzag, forking, nesting, parallel, orthogonal, centric, and single. This classification system was modified slightly in this study, and GA layouts have been classified as follows (Fig. 5):

- *Linear*: The intended reading order is indicated through the use of arrows and other visual cues. This structure effectively represents sequential processes such as experimental procedures and study results.
- *Zig-Zag*: This layout coordinates horizontal and vertical spatial dimensions to direct the viewer’s gaze via visual cues. It consists of multiple rows, and each row follows the same horizontal progression. Unlike the approach of Hullman and Bach (2018), which identifies zig-zag patterns across multiple graphics, this analysis focused on the internal layouts of individual graphics. Consequently, the number of such instances was relatively limited.
- *Forking*: In this layout, the structure diverges into multiple directions at least once within a linear flow (Fig. 6a); this category includes tree diagrams. This layout is particularly effective for representing complex processes or multifaceted relationships.

d. * Circular*: The elements are arranged in a circular pattern, and viewers are expected to view them sequentially (e.g., Noviello et al., 2021). This is useful for representing processes with steps that return to the beginning and repeat or where later stages influence the initial step.
e. *Detail*: A part of one element is connected to another using lines or shapes to indicate that the two are in an enlarged relationship. Hullman and Bach (2018) referred to this as “nesting” and stated that it was often used to incorporate representations that vary in the scale or level of detail; however, in this study this layout has been named as “detail” because this layout was used to indicate zooming (e.g., Nygaard et al., 2021).
f. *Axial*: One or two axes (horizontal or both horizontal and vertical) are used to present variables such as time or concentration, and the graphics or elements are arranged along these axes. This is similar to “orthogonal” layout (Hullman & Bach, 2018); however, owing to the existence of single-axis layouts, this layout has been termed as “axial” in this study (e.g., top graphic of the GA of Mittnenzweig et al., 2021). This layout is useful for describing changes along a temporal axis in a format that is distinct from a process chart, or it may be used to map items.
g. *Centric*: The central part contains the primary graphic, and supplementary information is arranged radially around it (e.g., Liu et al., 2021a, b, c). This format is useful for indicating that one element holds a higher priority and occupies a central position relative to the others.
h. *Parallel*: Multiple graphics or elements that are placed side by side without an explicit indication of the reading order (Fig. 6b). Although a parallel layout is convenient and accessible for article authors, readers may face difficulty in discerning the relationships among the individual elements in the graphics.
i. * Single*: This layout contains only one graphic (e.g., Kumon et al., 2021). By presenting only a single element, this format has the advantage of readily directing attention to that element.
j. *Model:* This format is frequently used in life sciences to depict models. Elements such as molecules are placed in specific cellular locations (e.g., the nucleus, mitochondria, or cytoplasm) because the positions themselves convey meaning and enumerate processes or relationships (e.g., Wang et al., 2021a, b).
k. *Tabular*: This format resembles a table with headings for each row and column. The elements or graphics are placed in the divided spaces (e.g., Liu et al., 2021a, b, c). This layout is as an effective means of representation of comparison of multiple elements.

Many GAs exhibited multilayered layouts with intergraphic and intragraphic layouts. Overall, the intergraphic layouts were generally not complex, and the most frequently used layouts were single or parallel. This study focused on the elements of the layouts within the graphics, and some graphics were subsequently classified as having multiple layouts. The results are summarized in Table 3.

**Table 3:** Analysis of GA layout

Layout	Number of graphics	Number of GAs	Percentage (GAs)
Linear	130	94	28.2%
Zig-Zag	4	4	1.2%
Forking	152	126	37.8%
Circular	12	11	3.3%
Detail	62	50	15.0%
Axial	41	38	11.4%
Centric	10	10	3.0%
Parallel	207	132	39.6%
Single	193	111	33.3%
Model	113	74	22.2%
Tabular	20	19	5.7%

“Linear,” “Zig-Zag” and “Forking” layouts were observed in 286 graphics (in a total of 224 GAs). They typically take the form of a flowchart connected by arrows (Fig. 6a). “Parallel” layouts were identified in 207 graphics (in 132 GAs) (Fig. 6b). The “Linear,” “Zig-Zag,” and “Forking” layout tended to be more prevalently used when the content type was “Method and context” or “Result explanation and model.” This prevalence may be attributed to the nature of these content types, which frequently requires the depiction of procedural flows and structural relationships essential to the study. In contrast, “Data image” tended to be depicted in “Parallel” layout.

### Representational Genre

**Fig. 7:**
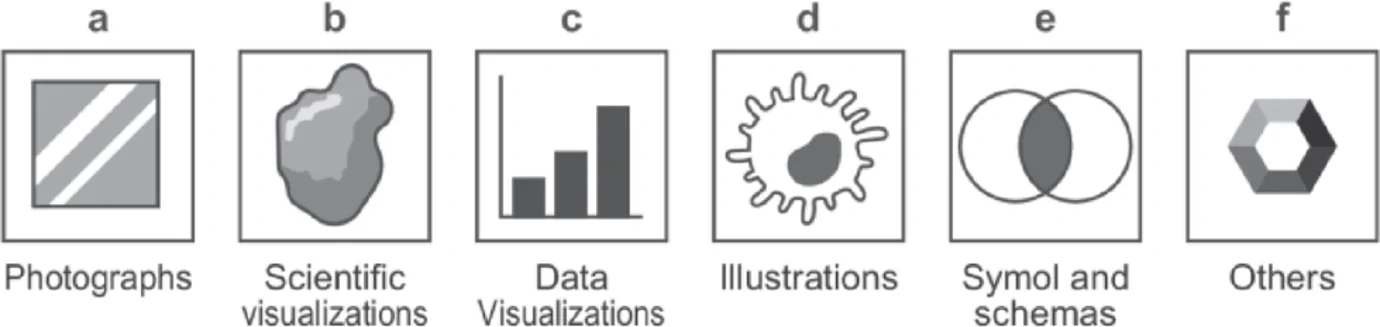
Six categories of representational genre of graphics

Hullman and Bach (2018) classified representational genres into photographs, scientific visualizations, illustrations, data visualizations, symbolic notations, and schemas. In this study, analysis of the samples showed that symbolic notation and schemas may be more appropriately combined into a single group as abstract representations. Additionally, the diagrams including pictorial elements were classified as “Illustrations.” Additionally, the category “Others” was introduced, and this category was not included as a genre classification in previous studies. These classifications are presented in Fig. 7.

**Fig. 8:**
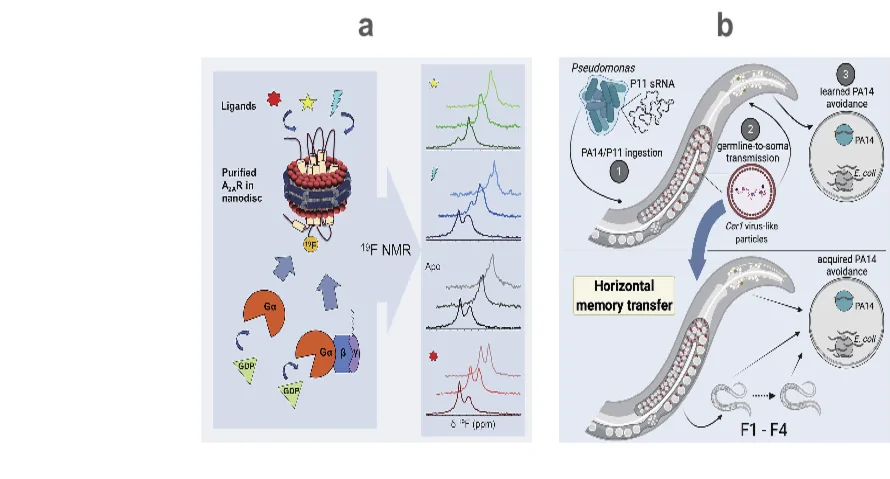
**a** Four graphics on the left of this GA are categorized as “data visualization” (Huang et al., 2021). They show fluorine nuclear magnetic resonance spectroscopy (19 F NMR) spectra of the A2A receptor, which illustrates the conformational ensemble in lipid bilayers.** b** This GA is an example of using illustrations in graphics (Moore et al., 2021). It presents a model showing how *C. elegans* utilizes Cer1 transposons for horizontal transfer of transgenerational memory

1. *Photographs*: These include photographs, microscopy and fluorescence microscopy images, and scanned images of experimental results (e.g., top left photos in the GA of Yu et al., 2021a, b). As direct representations of research findings, clear and intuitive photographs enhance the persuasive impact of arguments by providing visual evidence.
2. *Scientific visualizations*: Software generated 3D computer generated (CG) structures of proteins are placed under this classification (e.g., Nygaard et al., 2021). 3D CG protein structures are images reconstructed directly from data. Provided that the structure is sufficiently intuitive, these graphics enable a more accurate representation of the protein structure than if it were manually redrawn as an illustration.
3. *Data visualizations*: These include data or existing map information visualized as graphs or tables (Fig. 8a). Data visualizations potentially enhance the persuasive power of a GA, provided that the readers possess the necessary visual literacy and that the imagery permits an unambiguous interpretation.

d. *Illustrations*: This classification includes hand-drawn pictorial representation of objects (Fig. 8b). Illustrative expressions often enable a more rapid and intuitive understanding of contents than those offered by a textual description. In addition to effectively conveying interpretations and theoretical models, they function as an eye-catching element to attract the reader’s attention.
e. *Symbols and Schemas*: These include Venn diagrams or phylogenetic trees that represent abstract concepts (e.g., bottom graphic in the GA of Liu, Taassinari et al., 2021). Although symbols and schemas may be less intuitive than illustrations, they may significantly enhance comprehension by visually mapping the relationships between elements.
f. *Others*: Text-only, chemical formulas, and logos are classified here (e.g., top logo in the GA of Groussin et al., 2021). These formats may be useful when visual synthesis is challenging.

**Table 4:** Analysis of representational genre of graphics

Representational genre	Number of graphics	Number of GAs	Percentage (GAs)
Photographs	46	36	10.8%
Scientific visualizations	94	59	17.7%
Data visualizations	199	124	37.2%
Illustrations	643	305	91.6%
Symbol and schemas	49	41	12.3%
Others	21	19	5.7%

Table 4 shows the results of analysis of the representation genres of graphics. Illustrations were included in 643 graphics (in 305 GAs), which indicates that the majority of GAs incorporated pictorial representations. In this study, “illustrations” included not only drawn images but also visual languages such as pictograms. Most GAs included pictorial representations of biological entities (experimental animals or humans), organs or tissues (such as the brain), cells, and molecules. These illustrations either only represented the subject (e.g., a mouse) or additionally conveyed its state or condition (e.g., a dead mouse or clustered molecules).

Illustrations may resemble “very much the vernacular ways of expression or lying very close to the realistic appearances of things” (Dimopoulos et al., 2003, p. 191). In life science model diagrams, illustrative elements often take the form of diagrams connected by arrows and lines accompanied by textual labels or explanation (Abrahamsen et al., 2018). The major claims of articles may be conveyed effectively when illustrations combine conceptual or diagrammatic representations.

However, some graphics included experimental results directly in the form of photographs, scientific visualization, and data visualization perhaps because displaying experimental results may present the outcomes convincingly. However, photographs are used less frequently than scientific or data visualization. Despite the increasing use of photographs in research papers (Ariga & Tashiro, 2022), they are rarely used in GAs possibly because of the limited space in GAs and the tendency to express the primary message of the paper through models and text that interpret the photographs rather than thorough the photographs themselves.

### Originality

**Fig. 9:**
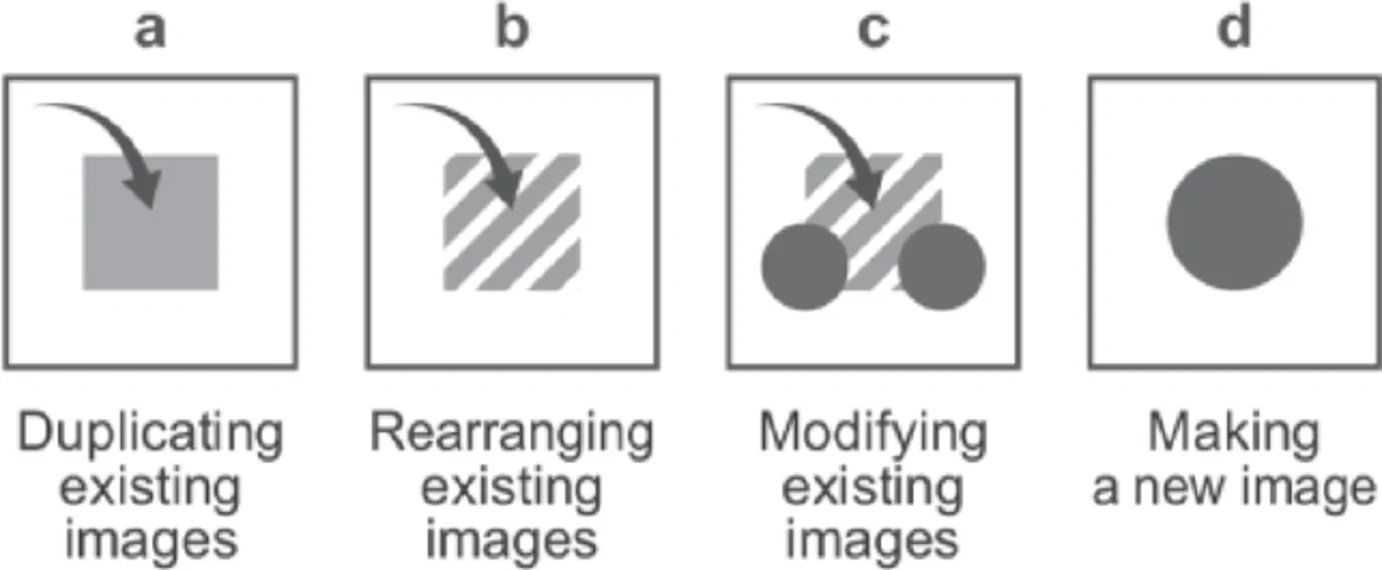
Four categories of originality of graphics

The GA guidelines of *Cell* instruct authors to differentiate between figures in the articles and GAs. To identify the frequency of duplication in life-science GAs, they were classified into the following categories (Fig. 9):

1. *Duplicating existing images*: Images from the paper were directly used in the GA with or without textual modifications. This method requires the least amount of labor for those creating GAs.
2. *Rearranging existing images*: Images from the paper were reused after adjusting the positions of the elements within the images. This approach is primarily used when constraint with structural layout or issues regarding contextual clarity within the GA preclude the direct inclusion of images in their original form.
3. *Modifying existing images*: Images from the paper were used with partial modifications such as changes in color or ratio or inclusion of additional elements. Although this requires more effort than the use of unmodified images, this method is effectively tailored to achieve visual integration with the rest of the GA.
4. *Making a new image*: Images were newly created in a different style from those in the paper or entirely new images were created. Although creating new images is the most labor-intensive approach, it significantly enhances the clarity and visual sophistication of the GA.

**Table 5:** Analysis of graphics originality

Originality	Number of graphics	Number of GAs	Percentage (GAs)
Duplicating existing images	53	36	10.8%
Rearranging existing images	74	53	15.9%
Modifying existing images	255	172	51.7%
Making a new image	448	222	66.7%

According to the results shown in Table 5, the most common pattern was creating a new image (222 GAs, 66.7%), followed by modifying existing images (172 GAs, 51.7%). Several researchers had attempted to redraw or create new images. Hendges and Florek (2019) analyzed 30 GAs in the fields of chemistry and engineering with focus on whether the GAs duplicated one or more existing images from the articles. They found that 13 GAs (43.3%) contained modified images, 9 GAs (30%) duplicated existing images, and 8 GAs (26.6%) contained entirely new images. Yoon and Chung (2017) analyzed 772 GAs in social sciences and reported that 58% of GAs duplicated images and only 7.38% contained new images. The results of the present study exhibit a trend that deviates from these previous reports, which suggests that researchers may have adhered to GA guidelines that discourage the reuse of images from the text.

In the context of the difference between GA images and figures in the articles, Ariga and Tashiro (2022) have stated that the figures in *Cell* articles predominantly display data with fewer illustrations explaining models or claims. In contrast, GAs frequently depict methods, data, and models, with particular emphasis on result explanation and models. This indicates that although the main text lacks a model or images explaining the result, many researchers have created new illustrations for the GAs. This trend suggests that the advent of GAs is accelerating the culture of visual explanation through illustrations.

Additionally, the figures in the articles were redrawn for some GAs in a different style and at higher quality (e.g., Chen et al., 2021). In many cases, the design and aesthetic quality of the GA images were superior to those of the article figures. This implies that researchers may have invested significant time and effort in creating GAs or may have enlisted the help of professionals or individuals skilled in drawing. This shows that researchers as authors may likely feel that time and effort are worth investing in creating high-quality GAs.

### Style of illustration

**Fig. 10:**
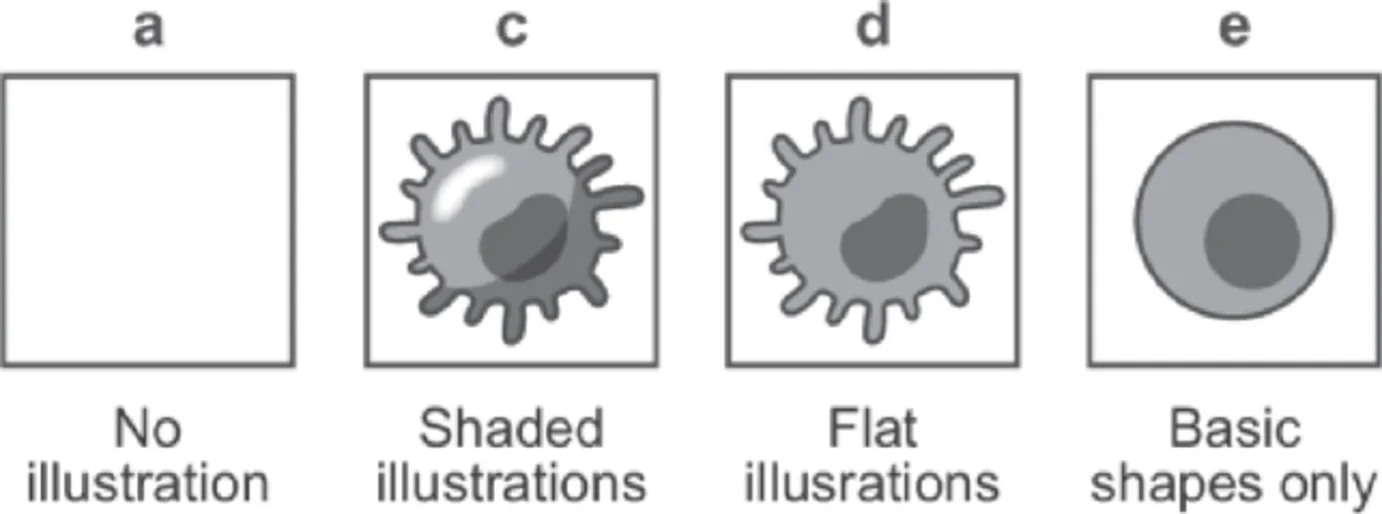
Four categories of illustration style of graphics

**Fig. 11:**
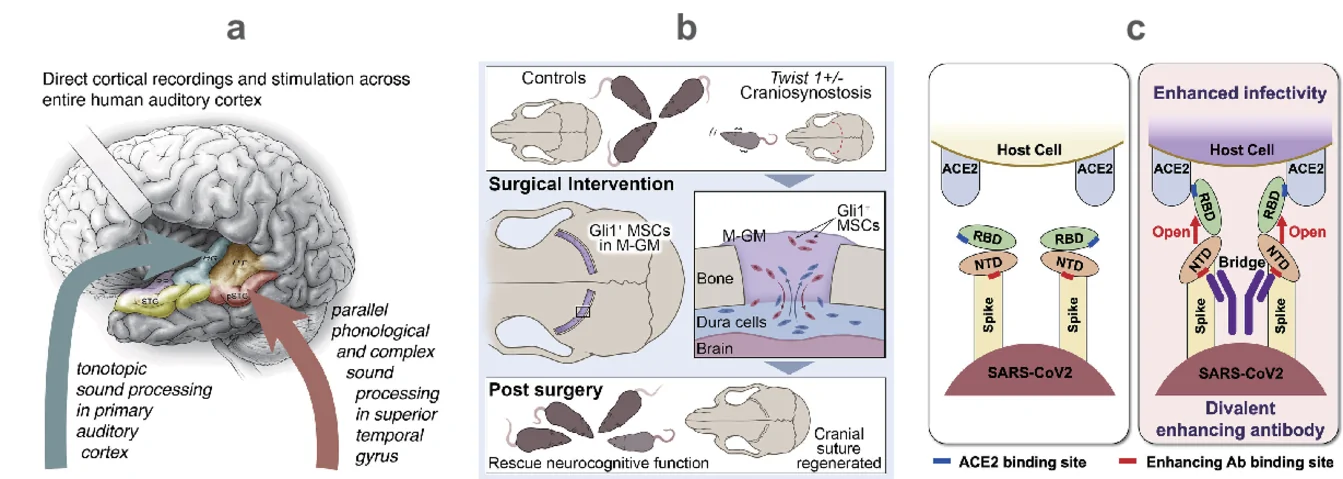
**a** This GA is an example of the use of shaded illustration (Hamilton et al., 2021). The GA depicts the parallel and distributed encoding of speech across human auditory cortex and features a shaded illustration of the brain to provide anatomical depth.** b** This GA is an example of using flat illustrations (Yu et al., 2021a, b). It outlines how cranial suture regeneration mitigates skull and neurocognitive defects in craniosynostosis by using flat illustrations to present the experimental methodology, proposed model, and key results.** c** This GA has been constructed using only basic shapes, and it illustrates an infectivity-enhancing site on the SARS-CoV-2 spike protein targeted by antibodies (Liu et al., 2021a, b, c).

The major styles of illustration in the GAs were analyzed and categorized as follows (Fig. 10):

1. *No illustration*: The graphics do not include illustrations (e.g., Noviello et al., 2021). This approach is effective when topics are difficult to depict visually or as a means to streamline the production process by eliminating the need to create or procure illustrative materials.
2. *Shaded illustrations*: The illustrations are not flat but include simple or complex shading to provide a three-dimensional appearance (Fig. 11a). This technique enhances the visual sophistication of the image and acts as an effective eye-catcher. This method is advantageous when using existing materials but is significantly more labor-intensive if the illustrations must be created from scratch.

c. *Flat illustrations*: These comprise simple fills and lines. The fills may be transparent, solid-colored, or simple gradients. This category includes pictograms and simple icons (Fig. 11b). They are easy to produce using software such as Adobe Illustrator. Furthermore, a consistent visual style may be maintained relatively easily even if the materials are sourced from different providers.
d. *Basic shapes only*: This category includes only rectangles, circles, straight lines, and arrows. These illustrations may be created easily using tools such as Adobe Illustrator or PowerPoint (Fig. 11c). However, using this approach for complex conceptual models may result in a presentation that lacks visual sophistication.

**Table 6:** Analysis of illustration style

Primary illustration style	Number of GAs	Percentage (GAs)
No illustration	23	6.9%
Shaded Illustrations	83	24.9%
Flat Illustrations	216	64.9%
Basic Shapes only	11	3.3%

As shown in Tables 6 and 216 GAs (64.9%) used the flat style of illustration created using Bézier curves. Icons and pictograms were frequently used, and some examples seem to use graphic tools such as Biorender (e.g., Rodda et al., 2021). Researchers were more likely to use images of scientific visualization than shaded illustrations in scenarios that focused on the complex structure of objects (e.g., Nygaard et al., 2021). The prevalence of flat and simple illustration styles may be attributed to low necessity, poor cost-effectiveness of detailed drawings, limited space availability, possible lack of artistic skills required for creating complex illustrations, and prevalence of flat-style illustrations in existing stock materials.

## Discussion

In summary, the analysis of the positioning of the GAs on the article page suggests that they are expected to function as a tool for readers to grasp the message of an article and decide whether or not to peruse the full text. GA guidelines indicate an expectation for readers to achieve immediate comprehension of the primary findings. Content analysis indicates that GAs tend to present result explanations and models with emphasis on the representation of the core research content. Additionally, several representational characteristics of GAs were identified such as the tendency to divide the visual space into multiple sections to accommodate numerous topics and frequent use of multilayered layouts with graphics nested within one another. These findings concur with those of previous studies that GAs aim to accelerate the understanding of the article and increase the overall interest and impact of the study. Illustrations are often rendered in a flat style, which may reflect limitations in time and skill required to produce shaded imagery and the widespread availability of flat-style assets in existing stock resources. Moreover, the relatively limited reuse of figures from the main text suggests that authors both adhere to journal guidelines and invest effort in producing GAs, possibly in anticipation of their communicative benefits.

This raises the question of how GA representations relate to other types of images. Among GA layouts such as linear, zig-zag, branching, circular, detailed, and model-based formats, visual elements such as arrows—referred to as glyphs by Abrahamsen et al. (2018)—are used, and this feature is shared with conventional mechanistic diagrams in life sciences. In fact, the observed instances of reuse further indicate a relationship between GAs and figures in research articles. However, several differences were also identified. GAs often incorporated multiple graphics within a single visual space and connected them through visual cues. Moreover, the main text tended to focus on data images, whereas GAs prioritized the graphical representation of interpretations such as conceptual models.

In this context, how do GAs relate to non-scientific visualizations? In this study, GAs have been inferred to align with the principles of modern infographics. An infographic is a representation of information or a method of visualizing information. Historically, this technique has been developed for visualizing statistical data such as graphs (Lankow et al., 2012). Currently, images that combine multiple charts and diagrams into a single narrative are widely used. These images often use background colors and flat simple pictograms and are designed to be aesthetically pleasing. The design is often completed in a single image, which encourages quick comprehension of information and facilitates sharing on social media. Infographic designers emphasize the presentation of visual information in an effective and efficient manner, and the method of designing is changing owing to digitalization (Santos et al., 2019).

One point of convergence between GAs and infographics is the practice of combining multiple graphics into a single image to create an overall narrative that is not typically observed in textbook illustrations. Additionally, background colors are frequently used in GAs to separate different graphics, which not a common trend in paper figures or textbooks. Additionally, the integration of flat-style illustrations with conceptual or schematic representations is a frequently used technique in infographics. Software commonly used for creating infographics such as Adobe Illustrator is also popular among researchers. Incidentally, articles that introduce infographic guides for creating GAs have been published (Spicer & Coleman, 2022).

Additionally, the emphasis on rapid comprehension inherent in infographics aligns with the tendency of GAs to present key findings concisely. This corresponds with the broader trend among academic journals to incorporate summaries and highlights that support the immediate understanding of research content (Hendges & Florek, 2019). Historically, scientific images such as botanical illustrations, data visualizations, and magnetic resonance imaging (MRI) have been used as records or evidence. Conceptual diagrams schematically convey scientific models. In contrast, GAs are “abstracts” of research articles where researchers focus on summarizing articles to enhance comprehension rather than engage in arguments, persuasion, or training. Both GAs and infographics have developed within a contemporary framework where the accessibility of digital applications intersects with an increasing demand for rapid intuitive information comprehension.

As previously mentioned, Daston and Galison (2007) analyzed the epistemic virtues of scientists through images. In contrast to the arguments of Daston and Galison, the influence on the representation of GAs may not be attributed solely to the authors. Although the authors and readers of GAs are often researchers, the journal managers are the ones who request the submission of GAs. In this case, the epistemic virtues and scientific self associated with GAs appear to be shared among authors, readers, and journal managers with subtle differences.

From the perspective of the article authors, the modern age is characterized by an increasing population of researchers and highly competitive information-saturated environment. Competition for the number of citations of research papers is fierce and is a fundamental aspect of academic evaluation (Hendges & Florek, 2019). In this context, authors may fear that their papers may be overlooked in a multitude of publications. Some researchers have argued that GAs should be seen as promotional tools for their work (Cox, 2015). In this era, researchers are under the scientific self of a surviving researcher and consequently invest in designs that can quickly and accurately convey information about their articles.

Similarly, GA readers are navigating an era of information overload with concerns of missing out on crucial information required for their research. Amidst the overwhelming number of papers that are being published, readers aim to instantly gather and understand information relevant to their own work. Hence, the readers are motivated by the same scientific self of a surviving researcher as that of the authors. Therefore, readers value the epistemic virtue of instant comprehension of papers for future publications.

Journal managers aim to achieve commercial success. Journals are facing a fear of survival owing to the rise of other commercial journals and open science. Consequently, they require authors to meet the demand of the readers for instant comprehension of information to gain authority and increase sales. Earlier, journals introduced summaries, highlights, and hyperlinks to support the readers’ need for immediate judgments. As mentioned previously, the introduction of GAs has reportedly increased the impact factors in some cases. The GA guidelines provided by *Cell* such as specific font sizes to improve article readability are additionally intended to ensure the proper functioning of the GAs. Thus, journals aim to survive by seeking to meet readers’ need for instant comprehension through visuals and establishing appropriate formats and rules for GAs. Hence, journal managers are key actors in shaping the visual culture of GA.

In this context, the epistemic virtue of instant comprehension in a society with an increasing number of papers and intense scientific competition is reinforced by all three actors involved in GAs, which promotes a new visual culture in science under the scientific self of the surviving researcher and journal.

## Conclusion

This study has investigated the visual culture created by GAs by focusing on their content, representation, and background. An analysis of the GAs in 333 articles published in *Cell* in 2021 shows that 295 GAs (88.6%) explained the results and models and succinctly presented the key findings. The primary aim of these images appeared to facilitate the understanding of the article’s main results and claims rather than to express background or significance. This study found that 251GAs (75.4%) used graphics to divide the area into two or three sections. Linear layouts were often used to illustrate processes, whereas parallel layouts were used to display data. The inclusion of multiple graphics within a single GA arises from the necessity to condense diverse information pertaining to an entire manuscript into a single visual frame. This structural complexity is considered a distinctive feature that distinguishes GAs from conventional figures found in research papers or textbooks. Illustrations were used in 643 graphics (305 GAs) to explain the methods and models. In total, 222 GAs (66.7%) were new graphics that were not found in the article. This trend toward labor-intensive production suggests that authors place high expectations on the functional role of GAs. The predominant style of these illustrations resembles the modern infographic design that emphasizes quick and clear communication of information online. These commonalities between GAs and infographics likely reflects the intense competition in the scientific community and the increase in the number of published papers. In this environment, the epistemic virtue of instant comprehension is reinforced by the three key actors associated with GAs: authors, readers, and journal managers. All three actors are driven by the scientific self of surviving researcher and journal.

This study does not specify who created the GAs. Some GAs indicate that authors use graphic makers such as Biorender. However, the exact methods and their proportions for GA creation remain unclear, and the extent to which these factors influence visual representation remains ambiguous. Thus, additional research is required. Furthermore, GAs may originate not only from manuscript figures but also from conference posters or presentation slides. As this study did not focus on individual researchers, the origins could not be traced beyond the main text of the paper. Future studies that focus on the investigators themselves and track the production process within the research cycle could potentially clarify these connections.

## Footnotes

1 Cell Press “Cell” https://www.cell.com/cell/home (Accessed July 10, 2024).

2 Elsevier “Cell” https://www.sciencedirect.com/journal/cell (Accessed July 10, 2024).

3 Cell Press “Cell Press Graphical Abstract Guidelines”. https://www.cell.com/pb/assets/raw/shared/figureguidelines/GA_guide-1537202744020.pdf (Accessed July 10, 2024).
